# ABO blood type correlates with survival in hepatocellular carcinoma following hepatectomy

**DOI:** 10.1038/s41598-017-04046-4

**Published:** 2017-06-30

**Authors:** Tao Wu, Xiao-An Ma, Guo-Qing Wang, Qing Li, Miao-Jing Li, Jin-Yue Guo, Xuan Liang, Zhi-Ping Ruan, Tao Tian, Ke-Jun Nan, Li-Na Liu, Hui Guo

**Affiliations:** 1 Department of Medical Oncology, The First Affiliated Hospital of Xi’an Jiaotong University, Xi’an, Shaanxi P. R. China; 2 Department of Gynecologic Tumor, Shaanxi Province Tumor Hospital, Xi’an, Shaanxi P. R. China; 3 Department of General Surgery, Xi’an central Hospital, Xi’an, Shaanxi P. R. China; 4 Department of Oncology, The Second People Hospital of Nanyang, Nanyang, Henan P. R. China

## Abstract

ABO blood types are associated with the prognosis of several malignancies. However, the role of the ABO blood type in hepatocellular carcinoma (HCC) remains elusive. Here, we evaluated the prognostic role of the ABO blood group in 691 HCC patients after hepatectomy by Cox regression analysis. A prognostic nomogram was generated to predict the 3 and 5-year overall survival (OS). A total of 262 HCC patients (37.9%) had blood group O, 199 (28.8%) had blood group A, 165 (23.9%) had blood group B, and 65 (9.4%) had blood group AB. The median OS was 55 months in patients with blood group O, 39 months for blood group A, 34 months for blood group B, and 34 months for blood group AB patients (*P* = 0.001, log-rank test). There were significant differences in OS between patients with blood groups O and A [hazard ratio (HR) = 1.416; 95% CI, 1.101–1.820; *P* = 0.007], blood group B (HR = 1.736; 95% CI, 1.333–2.262; *P* < 0.001), blood group AB (HR = 1.739; 95% CI, 1.210–2.499; *P* = 0.003) and non-O blood groups (HR = 1.485; 95% CI, 1.204–1.830; *P* < 0.001). Our constructed nomogram (c-index = 0.687) predicted the prognosis more accurately than the TNM stage alone(c-index = 0.601). In conclusion, non-O blood groups are poor prognostic indicators for HCC following hepatectomy. Our findings justify further external validation in larger cohorts.

## Introduction

Liver cancer is a frequent and highly aggressive malignancy. It is the third cause of cancer-related deaths worldwide^1^. Over 80% of liver cancers occur in less developed regions and 50% of all cases occur in China because of endemic hepatitis B virus (HBV) infection^2^. Notably, the incidence of liver cancer has increased in the United States and European countries over the past two decades^3, 4^. Hepatocellular carcinoma (HCC) represents the most common histological subtype of primary liver cancer, accounting for approximately 90% of cases^4^. HCC does not respond well to chemotherapy and is characterized by rapid progression and early vascular invasion. The long-term survival of patients with localized stage HCC can be improved by resection, transplantation and loco-regional therapies^5^. However, recurrence or distant metastases occur in most HCC patients and the 5-year survival rate is just 16%^6, 7^.

Prognostic factors are crucial for optimal therapy and disease surveillance. Established prognostic parameters such as stage, histological grade, and α-Fetoprotein (AFP) levels have been used in clinical practice^8^. However, the prognosis of HCC is often heterogeneous and therefore unsatisfactory^9^. Despite similar clinicopathological characteristics, HCC survival can be variable^10^. Therefore, new additional prognostic indicators should be identified to improve the outcome of surgically managed HCC patients.

Recently, the clinical implications of identifying the ABO blood type have extended beyond transfusion medicine^11^. The prognosis of HCC has been predicted using ABO blood typing, because this can be performed in every individual. Studies have shown that the ABO blood type is linked to the oncological outcome of various malignancies, including gastric cancer^12^, cervical cancer^13^, ovarian cancer^14^ and breast cancer^15^. A recent study reported that non-O blood groups increased the risk of HCC^16^. Another study indicated that blood group A increased the risk of developing HCC in a Korean population^17^. Although ABO blood type has been associated with the risk of HCC, its prognostic role in patients receiving hepatectomy for HCC is unclear.

In this study, we retrospectively evaluated the correlation of ABO blood type with prognosis in patients receiving hepatic resection as an initial treatment for HCC in a Chinese population. The results of this study showed that non-O blood groups (A, B, and AB) are poor prognostic indicators for HCC patients, suggesting that ABO blood typing status might represent a refined approach to prognostic stratification and individualized surveillance.

## Results

### Demographic data

A final total of 691 patients (564 males and 127 females) who received hepatic resection were included and completed follow-up. As shown in Table 1, the basic characteristics of patients were stratified by ABO blood type. The median age of participants was 54 years[interquartile range (IQR),25–75 years)]. The ABO blood types were as follows: O in 262 (37.9%) patients, A in 199 (28.8%) patients, B in 165 (23.9%) patients, and AB in 65 (9.4%) patients. Patient baseline characteristics were similar across the blood groups except for the AFP level, which was higher in individuals with blood group O(*P* = 0.014).

**Table1:** Patient clinicopathologic characteristics, stratified by ABO blood type.

Variable, no (%)	Over cohort	Blood group				*P*-value	Blood group		*P*-value
		O	A	B	AB		O	Non-O	
Total case	691	262 (37.9)	199 (28.8)	165 (23.9)	65 (9.4)		262 (37.9)	429 (62.1)	
Age (years)						0.882			0.525
<45	187 (27.1)	65 (24.8)	58 (29.1)	46 (27.9)	18 (27.7)		65 (24.8)	122 (28.4)	
45–60	308 (44.6)	118 (45.0)	83 (41.7)	77 (46.7)	30 (46.2)		118 (45.0)	190 (44.3)	
>60	196 (28.4)	79 (30.2)	58 (29.1)	42 (25.5)	17 (26.2)		79 (30.2)	117 (27.3)	
Sex						0.982			0.709
Male	564 (81.6)	212 (80.9)	163 (81.9)	136 (82.4)	53 (81.5)		212 (80.9)	352 (82.1)	
Female	127 (18.4)	50 (19.1)	36 (18.1)	29 (17.6)	12 (18.5)		50 (19.1)	77 (17.9)	
Place of residence						0.378			0.085
Rural	356 (51.5)	124 (47.3)	109 (54.8)	89 (53.9)	34 (52.3)		124(47.3)	232 (54.1)	
Urban	335 (48.5)	138 (52.7)	90 (45.2)	76 (46.1)	31 (47.7)		138 (52.7)	197 (45.9)	
Child-Pugh stage						0.209			0.127
A	648 (93.8)	241 (92.0)	192 (96.5)	153 (92.7)	62 (95.4)		241 (92.0)	407 (94.9)	
B	43 (6.2)	21 (8.0)	7 (3.5)	12 (7.3)	3 (4.6)		21 (8.0)	22 (5.1)	
Smoking						0.320			0.973
No	308 (44.6)	117 (46.0)	79 (39.7)	80 (48.5)	32 (49.2)		117 (46.0)	191 (44.5)	
Yes (former/current)	383 (55.4)	145 (55.3)	120 (60.3)	85 (51.5)	33 (50.8)		145 (55.3)	238 (55.4)	
Alcohol consumption						0.087			0.936
No	236 (34.2)	89 (34.0)	58 (29.1)	59 (35.8)	30 (46.2)		89 (34.0)	147 (34.3)	
Yes (former/current)	455 (65.8)	173 (66.0)	141 (70.9)	106 (64.2)	35 (53.8)		173 (66.0)	282 (65.7)	
Hepatitis status						0.954			0.414
HBV	538 (77.9)	197 (75.2)	159 (79.9)	130 (78.8)	52 (80.0)		197 (75.2)	341 (79.5)	
HCV	51 (7.4)	20 (7.6)	15 (7.5)	12 (7.3)	4 (6.2)		20 (7.6)	31 (7.2)	
HBV + HCV	7 (1.0)	2 (0.8)	2 (1.0)	2 (1.2)	1 (1.5)		2 (0.8)	5 (1.2)	
None	95 (13.7)	43 (16.4)	23 (11.6)	21 (12.7)	8 (12.3)		43 (16.4)	52 (12.1)	
Serum AFP level						0.014			0.003
<400 ng/ml	409 (59.2)	174 (66.4)	106 (53.3)	89 (53.9)	40 (61.5)		174 (66.4)	235 (54.8)	
>400 ng/ml	282 (40.8)	88 (33.6)	93 (46.7)	76 (46.1)	25 (38.5)		88 (33.6)	194 (45.2)	
Tumor differentiation						0.881			0.936
Well	118 (17.0)	43 (16.4)	38 (19.1)	28 (17.0)	9 (13.8)		43 (16.4)	75 (17.5)	
Moderate	413 (59.8)	158 (60.3)	117 (58.8)	101 (61.2)	37 (56.9)		158 (60.3)	255 (59.4)	
Poor	160 (23.2)	61 (23.3)	44 (22.1)	36 (21.8)	19 (29.3)		61 (23.3)	99 (23.1)	
Liver cirrhosis						0.846			0.719
No	191 (27.7)	76 (29.0)	56 (28.2)	41 (24.8)	18 (27.7)		76 (29.0)	115 (26.8)	
Yes	481 (69.6)	180 (68.7)	139 (69.8)	117 (71.0)	45 (69.2)		180 (68.7)	301 (70.2)	
Unknown	19 (2.7)	6 (2.3)	4 (2.0)	7 (4.2)	2 (3.1)		6 (2.3)	13 (3.0)	
Tumor size						0.428			0.367
≤5 cm	362 (52.4)	143 (54.6)	95 (47.7)	87 (52.7)	37 (56.9)		143 (54.6)	219 (51.0)	
>5 cm	329 (47.6)	119 (45.4)	104 (52.3)	78 (47.3)	28 (43.1)		119 (45.4)	210 (49.0)	
AJCC TNM Stage						0.678			0.643
I	181 (26.2)	72 (27.5)	50 (25.1)	45 (27.3)	14 (21.5)		72 (27.5)	109 (25.4)	
II	287 (41.5)	103 (39.3)	79 (39.7)	74 (44.8)	31 (47.7)		103 (39.3)	184 (42.9)	
III	223 (32.3)	87 (33.2)	70 (35.2)	46 (27.9)	20 (30.8)		87 (33.2)	136 (31.7)	

Notes: AFP α-fetoprotein; HBV hepatitis B virus; HCV hepatitis C virus; TNM Tumor node metastasis.

### Correlation of ABO blood type with survival

**Figure 1:**
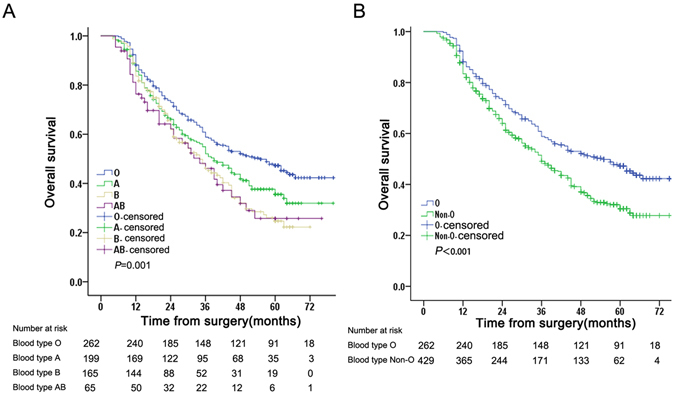
Overall survival (OS)for of HCC patients. (**A**). OS according to ABO blood types. (**B**). OS according to blood type O and non-O blood types.

**Figure 2:**
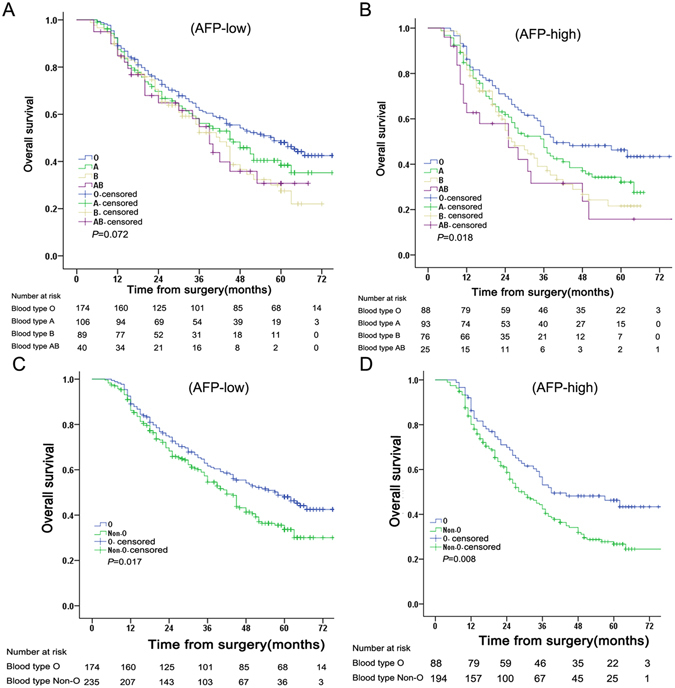
Overall survival (OS) of HCC patients according to α-fetoprotein (AFP). (**A**) OS of patients with low AFP levels according to ABO blood type. (**B**) OS of patients with high AFP levels according to ABO blood type; (**C**) OS of patients with low AFP levels according to blood type O and non-O blood types. (**D**) OS of patients with high AFP levels according to blood type O and non-O blood types.

Overall, 392 patients died and 299 survived after a median follow-up of 36 months (IQR, 25–75 months). As shown in Figure 1A, the blood group correlated significantly with OS (*P* = 0.001). The 1-year survival rate was 85.2%, the 3-year survival rate was 52.9%, and 5-year survival rate was 38.7%. The median OS times were 55 months (95% CI, 42.69–67.31 months) for blood group O, 39 months (95% CI, 32.20–45.80 months) for blood group A, 34 months (95% CI, 26.58–41.42 months) for blood group B, and 34 months (95% CI, 24.09–43.91 months) for blood group AB. The 1-, 3- and 5-year OS rates were markedly higher in HCC patients with blood group O than patients with other blood groups (Table 2). OS was similar in patients with A/B/AB blood groups (*P* = 0.226).Therefore, we divided the entire cohort into two subgroups [blood group O versus non-O blood groups (A, B, and AB)]. The median OS for patients with blood group O was 55 months (95% CI, 42.69–67.30 months) and non-O blood groups was 36 months (95% CI, 31.18–40.81 months). As seen in Figure 1B and Table 2, OS was significantly higher in patients with blood type O blood compared with non-O blood types (*P* < 0.001). Our findings indicated that blood group AB correlated positively with AFP levels. Thus, we further examined the interaction between blood type and AFP level as a predictor of prognosis. As shown in Figure 2, OS differed between patients with non-O and O blood groups subdivided by AFP level. Of note, ABO blood group correlated significantly with OS in HCC patients with high AFP levels (AFP > 400 ng/ml, *P* = 0.018), but not in HCC patients with low AFP levels (*P* = 0.072).

**Table 2:** Prognostic factors for overall survival by univariate analysis.

Variable	All patients				*P*-value
	Over cohort	1-year OS	3-years OS	5-years OS	
Age (years)					0.044
<45	187	92.5%	56.2%	35.9%	
45-60	308	92.8%	57.1%	39.8%	
>60	196	74.6%	51.1%	35.9%	
Sex					0.470
Male	564	85.7%	54.1%	38.0%	
Female	127	83.3%	47.9%	36.3%	
Place of residence					0.245
Rural	356	85.0%	49.3%	35.9%	
Urban	335	85.5%	56.7%	39.6%	
Child-Pugh stage					0.457
A	648	84.9%	55.3%	38.1%	
B	43	90.5%	47.0%	31.1%	
Smoking					0.668
No	308	84.6%	54.4%	36.5%	
Yes (former/current)	383	85.8%	53.3%	38.6%	
Alcohol consumption					0.968
No	236	86.6%	53.3%	36.2%	
Yes (former/current)	455	84.5%	52.8%	38.3%	
Hepatitis status					0.118
HBV	538	87.4%	54.8%	39.7%	
HCV	51	78.1%	56.4%	31.1%	
HBV + HCV	7	85.7%	64.3%	42.9%	
None	95	76.7%	45.0%	29.8%	
Serum AFP level					0.008
<400 ng/ml	409	87.4%	57.7%	40.7%	
>400 ng/ml	282	82.1%	45.9%	33.4%	
Tumor differentiation					<0.001
Well	118	94.1%	64.4%	50.8%	
Moderate	413	88.0%	55.5%	39.0%	
Poor	160	74.0%	37.6%	24.3%	
Liver cirrhosis					0.605
No	191	87.0%	54.9%	38.3%	
Yes	481	81.0%	48.8%	35.4%	
Unknown	19	84.2%	44.7%	-	
Tumor size					0.001
≤5 cm	362	88.2%	61.3%	41.8%	
>5 cm	329	82.0%	43.9%	33.1%	
AJCC TNM Stage					<0.001
I	181	93.9%	70.2%	52.9%	
II	287	86.6%	52.9%	36.3%	
III	223	76.4%	38.0%	25.9%	
ABO blood group					0.001
O	262	88.2%	58.8%	47.3%	
A	199	85.6%	52.1%	35.5%	
B	165	83.6%	48.0%	24.7%	
AB	65	76,3%	46.1%	25.7%	
ABO blood group					<0.001
O	262	88.2%	58.8%	47.3%	
Non-O	429	83.4%	49.1%	30.5%

Univariate analysis revealed that age (*P* = 0.044), serum AFP levels (*P* = 0.008), tumor size (*P* = 0.001), stage (*P* < 0.001), differentiation (*P* < 0.001), and ABO blood group (*P* = 0.001) were prognostic indicators for overall outcome. Further multivariate analysis identified age, tumor TNM stage and tumor differentiation as significant prognostic factors for OS. In particular, the ABO blood type was a prognostic indicator for OS. The hazard ratio (HR) of patients with blood type A was 1.416 (95% CI, 1.101–1.820, *P* = 0.007), the HR of patients with blood type B was 1.736 (95% CI, 1.333–2.262, *P* < 0.001), and the HR of patients with blood type AB was 1.739 (95% CI, 1.210–2.499, *P* = 0.006), when compared with blood group O. Patients with non-O blood groups had a worse survival (HR = 1.485; 95% CI, 1.204–1.830; *P* < 0.001). These analyses are presented in Table 3.

**Table 3:** Prognostic factors for overall survival by multivariate analysis.

Variable	Hazard ratio (HR)	95% confidence interval	*P*-value
Age (years)			0.014
<45	reference	—	
45-60	1.092	(0.851—1.401)	0.489
>60	1.458	(1.113—1.938)	0.007
Tumor differentiation			<0.001
Well	reference	—	
Moderate	1.213	(0.902—1.631)	0.202
Poor	1.833	(1.320—2.547)	<0.001
Tumor size			
≤5 cm	reference	—	
>5 cm	1.242	(1.013–1.523)	0.037
AJCC TNM Stage			<0.001
I	reference	—	
II	1.462	(1.119—1.910)	0.005
III	2.216	(1.605—2.815)	<0.001
Serum AFP level			
<400 ng/ml	reference	—	
>400 ng/ml	1.168	(0.948–1.439)	0.145
ABO blood group			<0.001
O	reference	—	
A	1.416	(1.101–1.820)	0.007
B	1.736	(1.333–2.262)	<0.001
AB	1.739	(1.210–2.499)	0.003
Non-O	1.485	(1.204–1.830)	<0.001

### Prognostic nomogram for OS

**Figure 3:**
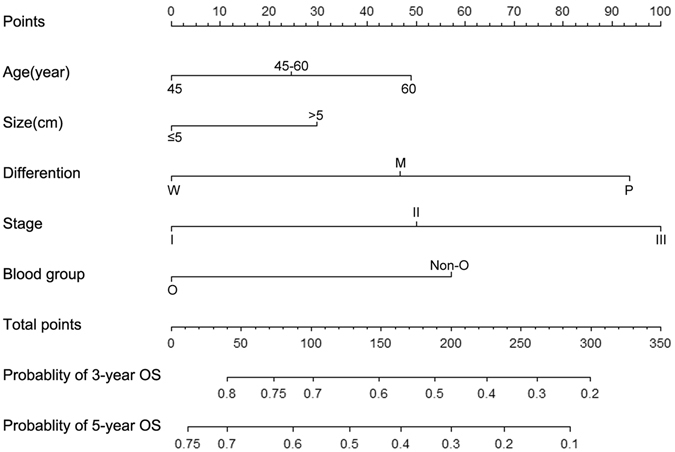
Nomogram predicting overall survival. Abbreviations: W, well; M, moderate; P, poor.

**Figure 4:**
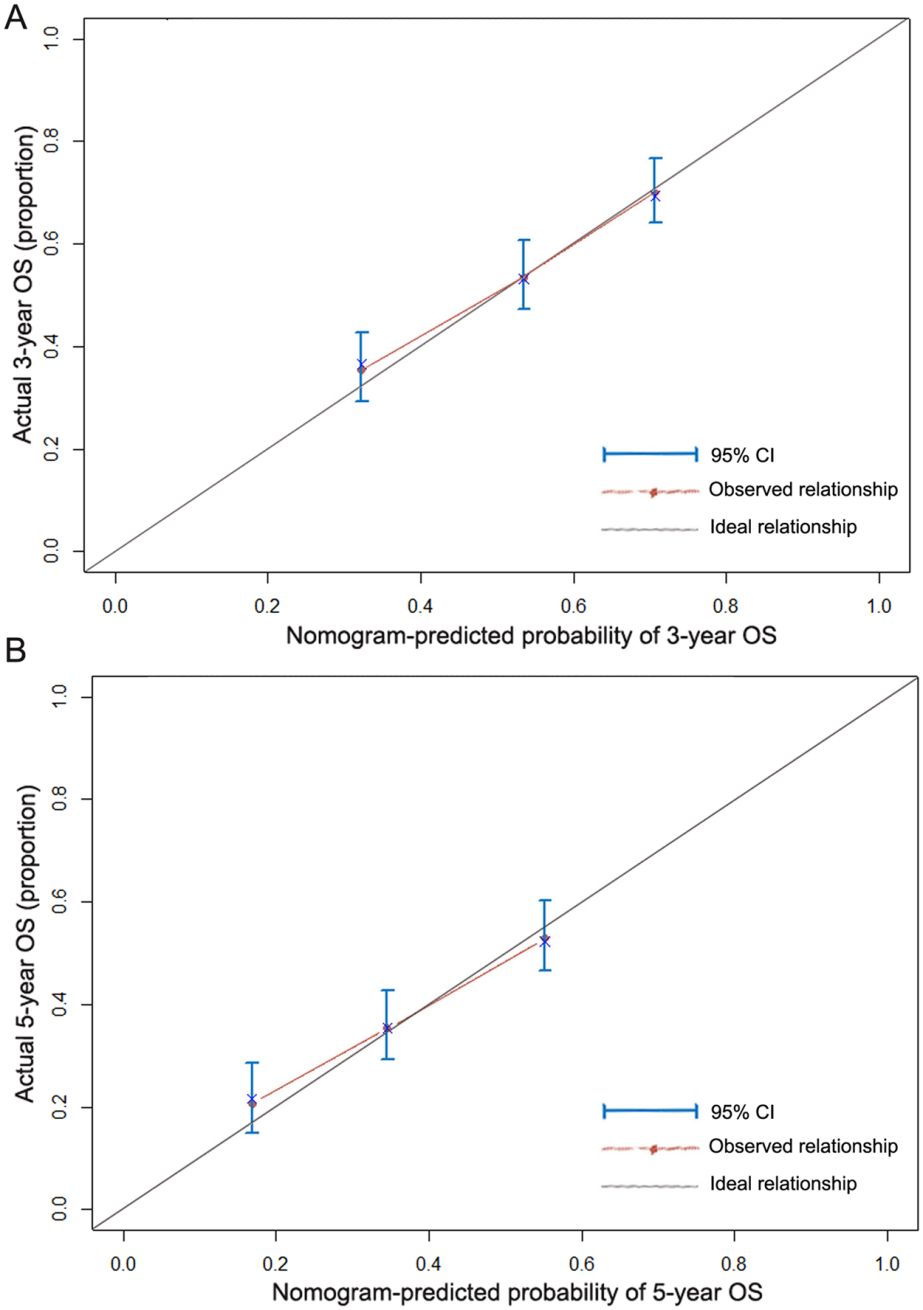
Calibration of nomogram-predicted 3-year overall survival (OS) (**A**) and 5-year OS (**B**).

As shown in Figure 3, we used a nomogram to predict the probability of death in individual HCC patients within 3 or 5 years after hepatectomy. Independent prognostic factors (age, tumor stage, differentiation and blood type) were incorporated into the nomogram. Calibration curves are shown in Figure 4, and these were similar to the ideal model. The Harrell’s c-index for OS was 0.687, higher than that for the TMN stage alone (0.601), This indicated that the nomogram was able to predict the prognosis more accurately.

## Discussion

ABO blood type was recently shown to affect the risk of multiple cancers^18, 19^, including HCC^16, 17^. Based on these reports, we hypothesized that the ABO blood group is associated with survival in HCC patients.

In the current study, ABO blood group was associated with OS in a large cohort of HCC patients following hepatectomy. The prognosis and overall outcome were worse in HCC patients with non-O blood type compared with blood group O. Interestingly, the ABO blood type did not correlate with OS in HCC patients with low AFP levels. The correlation between blood type and oncologic outcome of HCC has not been investigated so far. Our results are consistent with previous studies in patients with pancreatic cancer^20^, renal cancer and bladder cancer^21, 22^. These studies indicated a better prognosis in patients with blood type O compared with non-O blood groups.

Prognostic nomograms can evaluate the prognosis in individual patients^23^. In this study, we developed a novel nomogram incorporating ABO blood type and other prognostic factors. Our proposed nomogram was more discriminative than the TNM stage in predicting the prognosis of HCC after liver resection. In addition, our nomogram (model) might facilitate accurate prognostic stratification and the selection of optimal therapies. The serum blood type can easily be determined in all HCC patients and understanding the influence of blood group on tumor biology or therapeutic response may accelerate the development of individualized therapy for HCC.

The mechanisms by which ABO blood type influences HCC patient outcomes remains to be fully elucidated. However, several plausible hypotheses may explain the correlation. The *ABO gene* contains two alleles (A and B) that encode glycosyltransferases that catalyze the transfer of nucleotide donor sugars to the H antigen, which is then converted into ABO blood group antigens^24^. Aberrant glycosylation is a hallmark of cancer progression^25^. Terada and colleagues^26^ reported that ABO antigens are usually expressed in HCC tissue but not in normal liver and chronic hepatitis tissue. This suggests that alterations in glycosyltransferase are involved in HCC carcinogenesis and may explained how blood group affects OS in HCC patients. A correlation has also been reported between ABO blood group and liver disease. Poujol-Robert *et al*. revealed that individuals with non-O blood groups had an increased risk of liver fibrosis following HCV infection^27^. Moreover, Li *et al*. reported that non-O blood groups may increase the risk of HCC. Furthermore, a link between ABO and important cytokines has been shown, which may promote the development of HCC^28^. Recent studies have revealed single nucleotide polymorphisms in the ABO locus that correlate with circulating levels of tumor necrosis factor-alpha (TNF-a) and intercellular adhesion molecule-1(ICAM-1)^29, 30^. TNF-a is a multifunctional inflammatory cytokine involved in hepatocarcinogenesis. ICAM-1 regulates the immune response, which is implicated in the antigen-presenting mechanism^31^. Serum soluble ICAM-1(sICAM-1) has been associated with occurrence and prognosis of HCC^32^. Interestingly, a recent genomic study demonstrated that sICAM-1 levels are linked to ABO gene variants^33^. Patients with non-O blood groups express low levels of sICAM-1, compared with blood group O^34^, and reduced sICAM-1 levels may promote tumor metastasis in these patients^35^. These biological mechanisms may explain the favorable survival of HCC patients with blood type O. The mechanism underlying the interaction between blood type and AFP level is currently not well understood and should be further explored.

There were limitations to the present study. The major disadvantage of this analysis was its retrospective nature. The study was not representative of the general population and the possibility of selection bias cannot be ruled out. The ABO blood group was analyzed in relation to race, but all participants were Chinese; therefore these findings cannot reliably be extrapolated to other populations. The Eastern Cooperative Oncology Group scores were was not assessed because these data were not available. Furthermore, unidentified confounders may have biased our results. All participants had received a hepatectomy. Therefore, the findings cannot be used to predict associations between blood group and survival of patients with advanced HCC.

## Conclusions

In summary, we have shown that ABO blood type is associated with the prognosis of Chinese HCC patients after hepatic resection. OS is reduced in patients with non-O blood types (blood group A, B, and AB) compared with blood type O. Further studies are necessary to confirm these findings and to elucidate the underlying mechanisms.

## Patients and Methods

### Ethics statements and patients

The Institutional Review Board (IRB) of the First Affiliated Hospital of Xi’an Jiaotong University approved this study. The methods were performed in accordance with approved ethical guidelines. Informed consent was obtained from all eligible patients. After obtaining IRB approval, we retrospectively reviewed the medical records of patients who underwent hepatectomy at the First Affiliated Hospital of Xi’an Jiaotong University between 2008 and 2013. The exclusion criteria were as follows: previous anti-cancer treatment prior to surgical resection, vascular invasion, portal vein tumor thrombus or extra-hepatic spread, a class C Child-Pugh score of liver function, death within 30 days after surgery and poor data integrity. A total of 691 HCC patients were enrolled in the final analysis.

### Clinical variables

Factors potentially related to survival were collected, including age, gender, place of residence, liver cirrhosis, tumor size, differentiation, stage, Child-Pugh grades and hepatitis status. Pre-operative serum AFP levels were also included in analysis. Information about alcohol consumption and tobacco use was obtained from the patients. All tumor-related factors were determined by pathological examination of HCC tissue.

### Study endpoints and survival data

Outcome data were collected by telephone survey until October 2014 or until patient death. Overall survival (OS) was defined as the time from start of surgery to death from any cause or the last follow-up date. Patients alive at the time of last follow-up were defined as censored data.

### Statistical analysis

Categorical variables were compared using the Chi-square test or Fisher exact test. The cumulative OS was estimated according to the Kaplan-Meier method and tested using the log-rank test. The prognostic value of each factor was determined according to the Cox proportional hazards regression model. Significant prognostic indicators of endpoints in univariate analysis were included in the multivariate analysis. A nomogram was formulated based on the results of the multivariable analysis. Harrell’s c-index and calibration curve were used to assess the performance of the nomogram^36^. These activities were calculated using bootstrapping with 1000 repetitions. The prognostic nomogram were constructed and analyzed by R 3.3.1 with rms packages (http://www.r-project.org). Other statistical analyses were performed using SPSS23.0 software (IBM Corporation, Armonk, NY, USA). Statistical significance was set at P < 0.05.
